# Emotional demands at work and risk of hospital-treated depressive disorder in up to 1.6 million Danish employees: a prospective nationwide register-based cohort study

**DOI:** 10.5271/sjweh.4020

**Published:** 2022-04-29

**Authors:** Ida EH Madsen, Jeppe Karl Sørensen, Julie Eskildsen Bruun, Elisabeth Framke, Hermann Burr, Maria Melchior, Børge Sivertsen, Stephen Stansfeld, Mika Kivimäki, Reiner Rugulies

**Affiliations:** 1 National Research Centre for the Working Environment, Copenhagen, Denmark; 2 Unit Psychosocial factors and mental health, Federal Institute for Occupational Safety and Health, Berlin, Germany; 3 Sorbonne Université, Inserm, Institut Pierre-Louis d’Épidémiologie et de Santé Publique, IPLESP, Research Group in Social Epidemiology, Paris, France; 4 Department of Health Promotion, Norwegian Institute of Public Health, Bergen, Norway; 5 Department of Research and Innovation, Helse Fonna HF, Haugesund, Norway; 6 Department of Mental Health, Norwegian University of Science and Technology (NTNU), Trondheim, Norway; 7 Centre for Psychiatry, Barts and the London School of Medicine and Dentistry, Queen Mary University of London, London, UK; 8 Department of Public Health, Clinicum, Faculty of Medicine, University of Helsinki, Helsinki, Finland; 9 Finnish Institute of Occupational Health, Helsinki, Finland; 10 Department of Epidemiology and Public Health, University College of London, London, UK; 11 Department of Psychology, University of Copenhagen, Denmark; 12 Section of Epidemiology, Department of Public Health, University of Copenhagen, Denmark

**Keywords:** Denmark, depression, job exposure matrix, psychosocial, register-based study, stress

## Abstract

**Objective:**

Previous studies on effects of emotional demands on depression have relied on self-reported exposure data and lacked control for potential confounding by pre-employment risk factors for depression. This study used a register-based design to examine the risk of hospital-treated depressive disorder in relation to occupational levels of emotional demands at work, furthermore addressing the role of risk factors for depression before workforce entry.

**Methods:**

We analyzed data from two Danish register-based cohorts – Job Exposure Matrix Analyses of Psychosocial Factors and Healthy Ageing in Denmark (JEMPAD, N= 1 665 798) (17) and Danish Work Life Course Cohort (DaWCo, N=939 411), which link assessments of emotional demands by job exposure matrices to records of hospital-treated depressive disorder among employees aged 15–59 years at baseline (average follow up: 9.7 years in JEMPAD, 7.3 years in DaWCo). Potential confounders comprised sociodemographics, job control, work-related violence and physical demands at work. In DaWCo, we followed individuals from their entry into the workforce, and also included data on risk factors for depression before workforce entry (eg, parental income, education, and psychiatric diagnoses).

**Results:**

Employees in occupations with high emotional demands had an increased risk of hospital-treated depressive disorder with confounder-adjusted hazard ratios of 1.32 [95% confidence interval (CI) 1.24‒1.41] and 1.19 (95% CI 1.09‒1.30) in JEMPAD and DaWCO, respectively. This association remained after controlling for risk factors before workforce entry.

**Conclusions:**

This study suggests that employees in occupations with high emotional demands are at increased risk of hospital-treated depressive disorder. This increased risk was neither attributable to reporting bias nor explained by the included risk factors for depression recorded before workforce entry.

Emotional demands at work, ie, aspects of work that require sustained emotional effort (1), occur most frequently in occupations where a central part of the work tasks is the interaction with individuals from outside the workplace. Particularly, employees in human service occupations such as healthcare, education, or social work report high emotional demands at work (2). Such occupations place high demands on the employees to empathize with the patients, students or clients they work with, who may be in difficult, painful or otherwise distressing situations (3–5). Over time, this emotional work is thought to potentially lead to compassion fatigue and increase the risk of depression (6).

Observational studies have linked high emotional demands at work with an increased risk of depressive disorder, ascertained by a psychiatric interview (7), hospital-treatment (8), treatment with antidepressants (2), or a self-administered rating scale (9). A recent review and meta-analysis on psychosocial stressors at work and risk of depressive disorders reported a pooled risk of 1.21 (95% CI 1.08–1.36) for emotional demands (10).

However, questions have been raised regarding the causality of the association. One study (11) found that emotional demands at work predicted depressive disorder when measured with items relating to the overall subjective perception of emotional demands (eg, “Is your work emotionally demanding?” but not when examining task-specific emotional demands measured with more factual and less subjective questions (eg, “Do you have to care for the emotional needs of others?”). This finding suggests that the association between emotional demands and depressive disorder may be inflated by the self-reported exposure measurement, as the reported level of emotional demands may be influenced by the affective state of the respondent (reporting bias), a long standing methodological concern in psychosocial work environment research (10, 12).

Another concern is that previous studies have not accounted for potential confounding by other risk factors for depressive disorder, which may be unequally distributed between employees with high compared to low emotional demands due to the selection of individuals at risk of depressive disorder into occupations with high emotional demands. Such selection may be related to processes motivating young individuals to enter care work professions such as higher levels of childhood parentification (ie, excessively taking care of other family members’ needs) (13, 14), eg, due to parental illness or socioeconomic difficulties. This possible confounding by pre-employment risk factors has been illustrated by a study showing increased antidepressant treatment in care work professionals years before entering their profession (15) and another study showing higher levels of emotional demands in employees who have suffered from mental illness in childhood or adolescence (16).

Accordingly, the present study aimed to (i) examine the longitudinal association between emotional demands and depressive disorder using an exposure measurement that is not prone to reporting bias and (ii) control this association for depression risk factors present before workforce entry. To this end, we analyzed data from two Danish register-based cohorts: Job Exposure Matrix Analyses of Psychosocial Factors and Healthy Ageing in Denmark (JEMPAD) (17) and Danish Work Life Course Cohort (DaWCo) (18) which contain measures of emotional demands assessed by job exposure matrices for a large number of participants, in addition to measures of clinical diagnoses of depressive disorder through registers of psychiatric in- and outpatient hospital treatments.

## Methods

### Study design and populations

We chose to analyze data from the two Danish workforce cohorts, JEMPAD and DaWCo, in parallel, as they complement each other; while JEMPAD includes a wider age range of individuals (aged 30–59 years at baseline), DaWCo offers the possibility to control for risk factors of depressive disorder before workforce entry, by including data on parental socioeconomic position as well as psychiatric and somatic diagnoses, but includes only individuals aged 15–30 at baseline.

JEMPAD is a nationwide cohort with information on employment, psychosocial factors at work, health, labor market affiliation and socio-demographics. Details of JEMPAD have been published elsewhere (17). Briefly, JEMPAD included all employed individuals residing in Denmark in 2000, 30–59 years old, and with complete data on gender, age, and migration background, a total of 1 680 214 individuals. Using the unique Danish civil registration number, we linked these individuals to other population-based registers that provided information on socio-demographics, health services use, diagnoses for in- and out-patient hospital treatment, and causes of death. We excluded individuals with diagnosed depressive disorder before or in the year of baseline (N=14 516), yielding an analytic cohort of 1 665 798 individuals followed for 16 113 287 person years (mean follow up: 9.7 years).

DaWCo is an open inception cohort study of all individuals who first entered the Danish workforce during the years 1995–2009 and were 15–30 years old at workforce entry. Details of DaWCo have been published elsewhere (18). Briefly, DaWCo was constructed using population-based Danish registers on employment, health, demographic and socioeconomic factors to examine effects of working conditions on health. Working conditions were measured repeatedly throughout the work life by applying annually updated job exposure matrices. Workforce entry was defined as the first year with employment as the main source of income (N=979 257). We excluded individuals with missing data on gender and migration background (N=5 176), and individuals who died (N=71), emigrated (N=13 087), or received disability pension (N=361) in their year of entry, leaving 960 562 individuals in the cohort. To study incident depressive disorder, we further excluded individuals with diagnosed depressive disorder before or in the year of workforce entry (N=4989). To avoid overlap between the two cohorts, we further excluded 16 162 individuals from the DaWCo population who were potentially included in the JEMPAD population, as they entered the workforce during 1995–2000 at the age of 25–30 years. The analytic study population for DaWCo consisted of 939 411 individuals, followed for 6 825 523 person years (mean follow up: 7.3 years).

### Measurement of emotional demands

Emotional demands were measured using a job exposure matrix (JEM) estimating the average gender- and age-specific mean levels of emotional demands in occupations classified according to the Danish version of the International standard classification of occupations (DISCO-88) (19) [see supplementary material (www.sjweh.fi/article/4020) table S1 for details]. The JEM was constructed based on survey data from the Danish Work Environment Cohort Study collected in 2000 (N=8583, response rate 75.0%) and 2005 (N= 12 413, response rate 62.5%) (20). In the surveys, emotional demands were measured using a 3-item scale (supplementary table S2). We constructed the JEM from the survey data using best linear unbiased predictor (BLUP) estimates of the level of emotional demands as a function of a person’s DISCO-88 occupation, gender, age and year of data-collection (2000 or 2005). The models were based on data from 10 299 first-time respondents to either DWECS 2000 or 2005. Occupations with <5 survey respondents were collapsed at a less detailed level of the DISCO-coding with fewer digits resulting in a JEM covering 246 occupations coded with 4 digits (210 occupations), 3 digits (22 occupations), 2 digits (12 occupations) or 1 digit (2 occupations). For further details on the JEM construction, see (21).

Each individual was assigned an annual level of emotional demands during 2000–2009 for JEMPAD and 1995–2009 for DaWCo, which was subsequently categorized by distribution quartiles in the JEMPAD population. In years of non-employment (eg, unemployment or studying), emotional demands were categorized as low. Supplementary table S3 shows the occupations with the highest and lowest levels of emotional demands.

### Measurement of depressive disorder

Information on depressive disorder was obtained from the Psychiatric Central Research Register (22) during 1969–1994 and the National Patient Register during 1995–2010 (23). These registers encompass all inpatient psychiatric admissions in Denmark since 1969 and from 1995 onwards also outpatient admissions (22). We defined depressive disorder as a main diagnosis of F32 or F33 from ICD-10 (for 1994–2010), and 296.0, 296.2, 298.0, 300.4 from ICD-8 (for 1969–1993). ICD-9 was never used in Denmark. To exclude individuals with depressive disorder prior to workforce entry, we additionally used codes F92.0 (ICD-10) and 308.02 (ICD-8) for depressive disorder in childhood or adolescence.

### Measurement of potential confounders

For both cohorts we included information on calendar year, gender, age, cohabitation, employment status, migration background, income, number of health services used, job control, risk of work-related violence, and physical demands at work, and any psychiatric diagnosis before study baseline (2000 for JEMPAD, workforce entry for DaWCo). Furthermore, for DaWCo, additional data were available, including information on childhood socioeconomic position, maternal and paternal psychiatric and somatic diagnoses before the cohort member entered the workforce. The choice of potential confounders was guided by existing literature indicating an increased risk of depression in relation to these factors (24–27) and considerations regarding their relationship with emotional demands at work.

Supplementary table S1 summarizes the measurement of potential confounders. All sociodemographic covariates were derived from Danish National Registers (22, 23, 28–31). For income, we used household income in the JEMPAD population but personal income in the younger DaWCo population, where many were likely still living with their parents in their first years in the cohort. Data on the number of health services used were from the Danish National Health services register, encompassing mainly primary health care services (31). To avoid adjustment for an intermediate step in a causal pathway, we included health services use data from the year preceding the year for measurement of exposure.

Job control, risk of work-related violence, and physical demands at work were measured using JEM. Job control and physical demands at work were categorized by the distribution quartiles of the JEMPAD population. Risk of work-related violence (yes/no) was dichotomized by predicted risk of work-related violence of ≥2%. This cut-off point was based on the distribution of the DaWCo population, where it distinguishes the upper quartile from the three lower quartiles. The correlations between the included working conditions are reported in supplementary table S4.

To measure psychiatric diagnoses before study baseline, we included diagnoses coded as chapter F (ICD-10) or 290-315 (ICD-8) recorded as main or subsidiary diagnoses during the baseline year or before.

Childhood socioeconomic position was measured in DaWCo by maternal and paternal employment status, education and income when the cohort member was 15 years. If information was missing, we included data from preceding years back to the birth of the cohort member and, if still missing, we included data up until age 20. Maternal and paternal psychiatric and somatic diagnoses were included from the Psychiatric Central Research Register and National Patient Register including both main and subsidiary diagnoses recorded before the cohort member entered the workforce. Linkage to parental data was available from 1980 onwards and only for individuals with parents residing in Denmark. To ensure cohort completeness, individuals with missing data were assigned to a separate category for those variables and retained in the analyses. Table S5 shows the distribution of individuals in relation to the included pre-employment risk factors for depression.

Age, cohabitation, employment status, income, health services use, and working conditions were included as annual time-varying variables, while all the remaining covariates were considered as time-invariant variables.

### Statistical analysis

We analyzed data using Cox proportional hazard regression models with time-to-first diagnosis of depressive disorder as the outcome. Fulfillment of the proportional hazards assumption was assessed by visual inspection of the log-log hazard plots. We used calendar time as the time-axis to account for period effects on psychiatric treatment (22). We analyzed data longitudinally with a one year time-lag, relating exposure during year t to events during year t+1. Individuals were followed from baseline (1 January 2000 in JEMPAD and date of workforce entry in DaWCo) until first depressive disorder diagnosis, death, emigration, receipt of disability pension, or end of follow-up (31 December 2010), whichever came first. We terminated follow-up in the year 2010 because exposures could not be updated after 2009 due to changes in the occupational classification.

In the main analysis, we adjusted for gender, age, cohabitation, employment status, migration background, income, health services use in the preceding year (t-1) in model 1. Model 2 was additionally adjusted for job control, work-related violence, and physical demands at work. For DaWCo, we included a model 3 additionally adjusting for the presence of a psychiatric disorder before workforce entry (yes/no), childhood socioeconomic position (maternal and paternal employment status, maternal and paternal education, and maternal and paternal income), and maternal and paternal psychiatric and somatic diagnoses. The applied analytic framework was based on considerations concerning the potential causal effects and temporal order of the included variables and is illustrated in our Directed Acyclic Graph in figure S1. We included other work-related factors in model 2 as a separate step because the direction between emotional demands and theses exposures is uncertain, whereas there is more certainty regarding the role of the variables included in model 1 as potential confounders.

As supplementary analyses, we examined the association between emotional demands and depressive disorder separately in men and women. We also examined the association between emotional demands and depressive disorder after excluding individuals with any diagnosed psychiatric disorder before baseline (JEMPAD, N=43 686) or workforce entry (DaWCo, N=48 303). In a post hoc analysis, we further investigated whether the unexpected direction of the association in model 1 for DaWCo was related to the categorization of emotional demands, by classifying the exposure according to the quartiles of the DaWCo population, rather than quartiles of the JEMPAD population. Finally, we conducted a quantitative bias analysis to estimate the extent of bias caused by non-differential misclassification of exposure by the JEM for emotional demands. This analysis accounts for the imperfect measurement of emotional demands due to the application of a JEM, and assumes that the measurement error is similar in high and low exposure groups and for individuals who become cases and for those who do not become cases. The analysis was conducted using the methods for quantitative bias analysis proposed by Lash, Fox, & Fink (32) using the spreadsheet developed for correcting analyses for exposure misclassification available online (https://sites.google.com/site/biasanalysis). We applied a sensitivity of 0.53 and a specificity of 0.87 based on data from the JEM construction. All statistical analyses were conducted in SAS version 9.4 (SAS Institute, Cary, NC, USA).

## Results

Table 1 shows the characteristics of the study populations at baseline (2000) for JEMPAD and in the year of workforce entry for DaWCo. Both populations were gender balanced, with about 50% women. The mean age was 44 years in JEMPAD and 20 years in DaWCo. The members of the DaWCo cohort tended to be more likely to work in occupations with low emotional demands, low job control and high physical demands, compared to the JEMPAD population.

**Table 1:** Baseline characteristics of the study populations.

	JEMPAD ^a^			DaWCo ^a^		
	
	N	%	Mean	N	%	Mean
Total sample	1 665 798	100	939 411	100	
Gender						
Men	860 073	51.6		476 400	50.7	
Women	805 725	48.4		463 011	49.3	
Age (years)			43.8			20.1
15–17				103 410	11.0	
18–19				379 211	40.4	
20–24				378 299	40.3	
25–30				78 491	8.4	
30–35	360 971	21.7				
36–45	587 774	35.3				
46–55	550 352	33.0				
56–60	166 701	10.0				
Cohabitation						
Yes, living with partner or spouse	1 238 902	74.4		606 184	64.5	
No, single	423 283	25.4		292 753	31.2	
Unknown	3 613	0.2		40 474	4.3	
Migration background						
No	1 590 543	95.5		804 526	85.6	
Yes	75 255	4.5		134 885	14.4	
Education						
Primary or lower secondary	387 832	23.3		684 674	72.9	
Upper secondary	764 149	45.9		167 502	17.8	
Short cycle tertiary	82 041	4.9		3617	0.4	
Bachelor or equivalent	290 729	17.5		11 285	1.2	
Master or equivalent	110 171	6.6		3 819	0.4	
Doctoral or equivalent	6 687	0.4		12	<0.1	
Unknown	23 698	1.4		68 502	7.3	
Annual disposable income DKK ^b^			320 591			101 087
Health services (N)			15.3			10.6
Any psychiatric diagnosis before baseline	43 686	2.6		48 303	5.1	
Emotional demands						
Low	406 982	24.4		754 938	80.4	
Medium - low	428 309	25.7		47 980	5.1	
Medium - high	415 479	24.9		104 223	11.1	
High	415 028	24.9		32 270	3.4	
Job control						
Low	421 253	25.6		722 697	76.9	
Medium - low	445 990	26.8		121 778	13.0	
Medium - high	383 293	23.0		75 575	8.0	
High	415 262	24.9		19 361	2.1	
Risk of work-related violence						
Low	1 276 370	76.6		751 761	80.0	
High	389 428	23.4		187 650	20.0	
Physical demands						
Low	394 550	23.7		14 273	1.5	
Medium - low	400 085	24.0		51 656	5.5	
Medium - high	450 667	27.1		316 698	33.7	
igh	420 496	25.2		556 784	59.3	

a Baseline year for JEMPAD cohort members is 2000 and for DaWCo cohort members their year of workforce entry.

b We measured income (Danish kroner) as household income in JEMPAD and personal income in DaWCo because many individuals in DaWCo shared households with their parents in the baseline year.

There were 22 378 individuals who became cases with depressive disorder in JEMPAD and 15 753 became cases in DaWCo. Amongst the cases, the majority (69.4% in JEMPAD, 74.4% in DaWCo) were diagnosed with F32 “Depressive Episode” (data not shown). Table 2 shows the associations between emotional demands and depressive disorder in the two populations under study. In model 1, employees in occupations with the highest level of emotional demands had a hazard ratio (HR) of depressive disorder of 1.24 [95% confidence interval (CI) 1.18‒1.30] in JEMPAD and 0.86 (95% CI 0.81‒0.92) in DaWCo. After adjusting for other working conditions (model 2), the association became more pronounced in JEMPAD with a HR of 1.32 (1.24‒‒.41). In DaWCo, adjusting for other working conditions reversed the association from model 1 and high emotional demands were now also associated with an increased risk of depression with a HR of 1.19 (95% CI 1.09‒1.30). There was no evidence of a dose–response association. In JEMPAD, the risk of depressive disorder amongst employees in occupations with medium levels of emotional demands was similar to the risk amongst employees with low emotional demands and, in DaWCo, the risk of depressive disorder was similar in employees with high and medium–high emotional demands. When further adjusting for risk factors for depressive disorder that pre-existed workforce entry in DaWCo (model 3), the results remained unchanged.

**Table 2:** Association between emotional demands and subsequent onset of depression in the JEMPAD and DaWCo cohorts. [CI=confidence interval; HR=hazard ratio; PY=person years.]

	PY	Cases	Cases per 10 000 PY	Model 1^a^			Model 2 ^b^			Model 3 ^c^		
		
				HR	95% CI	P-value	HR	95% CI	P-value	HR	95% CI	P-value
JEMPAD												
Emotional demands						<0.001			<0.001			
Low (reference)	5 405 126	8 878	16	1.00			1.00					
Medium - low	3 564 817	4 292	12	1.00	0.96‒1.05		1.07	1.01–1.12				
Medium - high	3 598 007	3 819	11	0.90	0.86‒0.95		1.02	0.96‒1.08				
High	3 545 337	5 389	15	1.24	1.18‒1.30		1.32	1.24‒1.41				
DaWCo												
Emotional demands						<0.001			<0.001			<0.001
Low (reference)	4 770 523	11 066	23	1.00			1.00			1.00		
Medium - low	670 191	1326	20	0.81	0.76‒0.86		0.94	0.88‒1.01		0.95	0.89‒1.02	
Medium - high	774 719	1898	25	1.00	0.95‒1.05		1.21	1.13‒1.30		1.20	1.12‒1.29	
High	610 090	1463	24	0.86	0.81‒0.92		1.19	1.09‒1.30		1.17	1.07‒1.27	

a Adjusted for gender, age, migration background, cohabitation, employment status, health services use, and income.

b Adjusted for model 1 + job control, work related violence and physical demands at work.

c Adjusted for model 2 + psychiatric diagnosis before workforce entry, maternal and paternal employment status, education, income, and maternal and paternal psychiatric and somatic diagnoses.

When analyzing men and women separately, we found that the association between high emotional demands and depressive disorder was largely similar between the genders in both cohorts (table 3). After excluding individuals with any psychiatric diagnosis before workforce entry from the DaWCo cohort, the associations became more pronounced with a HR of 1.29 (95% CI 1.14‒1.45) for employees in occupations with high emotional demands (supplementary table S6). In JEMPAD, results were similar to those from the main analysis after the exclusion of individuals with any psychiatric diagnosis before baseline (table S6).

**Table 3:** Association between emotional demands and subsequent onset of depression in the JEMPAD and DaWCo cohorts, for men and women separately. [CI=confidence interval; HR=hazard ratio; PY=person years.]

	PY	Cases	Cases per 10 000 PY	HR	95% CI	P-value
JEMPAD ^a^						
Men						
Emotional demands						<0.001
Low (reference)	3 813 939	5 455	14	1.00	
Medium - low	2 074 823	2 314	11	1.06	1.00–1.14	
Medium - high	1 478 224	1 246	8	0.97	0.89–1.05	
High	914 289	1 200	13	1.35	1.23–1.48	
Women						
Emotional demands						<0.001
Low (reference)	1 591 187	3 423	22	1.00	
Medium - low	1 489 995	1 978	13	1.04	0.95–1.14	
Medium - high	2 119 784	2 573	12	1.01	0.92–1.12	
High	2 631 048	4 189	16	1.28	1.15–1.42	
DaWCo ^b^						
Men						
Emotional demands						<0.001
Low (reference)	2 920 160	4 586	16	1.00	
Medium - low	197 405	160	8	0.87	0.73–1.04	
Medium - high	253 129	353	14	1.29	1.11–1.50	
High	108 308	127	12	1.18	0.94–1.50	
Women						<0.001
Emotional demands						
Low (reference)	1 850 363	6 480	35	1.00	
Medium - low	472 785	1 166	25	0.97	0.89–1.04	
Medium - high	521 590	1 545	30	1.16	1.07–1.24	
High	501 782	1 336	27	1.20	1.09–1.34	

a We report fully adjusted estimates, ie, for JEMPAD associations are adjusted for: age, cohabitation, employment status, migration background, income, health services use, job control, work related violence and physical demands at work.

b We report fully adjusted estimates, ie, for DaWCo associations are adjusted for age, cohabitation, employment status, migration background, income, health services use, job control, work related violence and physical demands at work, psychiatric diagnosis before workforce entry, maternal and paternal employment status, education, income, and maternal and paternal psychiatric and somatic diagnoses.

In a post-hoc analysis, we explored the unexpected finding that, in model 1 for DaWCo, high emotional demands were associated with a decreased risk of depressive disorder. When we changed the categorization of emotional demands in DaWCo to reflect quartiles of DaWCo, we found associations similar to those of our main analyses with a HR for model 1 of 0.91 (95% CI 0.85–0.97) for employees in occupations with high emotional demands, and HR of 0.87 (95% CI 0.81‒0.93) and 1.01 (95% CI 0.95–1.07) for employees in occupations with medium–high and medium–low emotional demands, respectively (data not shown). The quantitative bias analysis showed that when we accounted for the misclassification of exposure introduced by the JEM, the HR of 1.31 from JEMPAD increased to 1.90 (data not shown).

## Discussion

Using data from two independent Danish register-based cohorts, this study found that employees in occupations with high levels of emotional demands are at increased risk of depressive disorder. An association was observed in the JEMPAD population in both minimally and multivariably adjusted models, whereas the association was masked before adjusting for other working conditions (low job control, work-related violence and high physical demands at work) in the younger DaWCo population. This difference in results between the cohorts is likely explained by differing distributions of other working conditions. Members of DaWCo were more likely than members of JEMPAD to work in occupations with low control (76.9% versus 25.6% at baseline). Previous analyses have shown that low control predicts depression in DaWCo (25), and we found a positive correlation between emotional demands and job control (table S4), ie, individuals in occupations with higher levels of emotional demands on average worked in occupations with higher levels of job control. Consequently, in the model where we did not adjust the association between emotional demands and depression for the average higher levels of job control associated with higher emotional demands, the association between emotional demands and depression was masked. There are also other possible differences between the cohorts that could affect the potential effects of their working conditions on mental health. DaWCo members were all working during their year of cohort entry, but over time a proportion became students for a period until returning into employment (18). It is possible that the jobs held by DaWCo members in their early years differ to the jobs of JEMPAD members in terms of working part-time versus full-time, or the younger DaWCo members could be more likely to have a more short-term time perspective on their current position (33).

The association between emotional demands and depressive disorder was similar in men and women and did not appear to be explained neither by confounding by sociodemographic factors such as gender, age, income, nor by other working conditions such as job control, violence or physical demands at work, or by the included pre-employment risk factors for depressive disorder included in the DaWCo population. Furthermore, our quantitative bias analysis suggested that the true association between emotional demands and depression may be considerably stronger than that shown in the present paper, due to the misclassification of exposure introduced by the JEM. Consequently, the results of the present paper could be considered conservative estimates of the association between emotional demands and depression. While we included a range of confounders in our analyses – the choice of which was guided by considerations regarding their association to the examined outcome and potential relation to the exposure – we did not include analyses of effect modification. This type of analyses remains an important topic for further research.

### Comparison with previous studies

The present results add to previous studies linking high emotional demands at work with an increased risk of depressive disorder, ascertained by a psychiatric interview (7), hospital-treatment (8), treatment with antidepressants (2), or a self-administered rating scale (9). However, previous findings have been mixed, when comparing measures of emotional demands that are subjective in nature such as employees’ perceived emotional demands, to measures that are less subjective, such as measures focusing on the content of work tasks, and thus considered less prone to be affected by reporting bias (11). A recent study comparing different assessments of emotional demands in relation to risk of long-term sickness absence found that both content-related and perceived emotional demands were associated with an increased risk (34). Furthermore, that study found that while the association for perceived emotional demands was attenuated with adjustment for baseline depressive symptoms, the association for content related emotional demands remained largely unchanged. Our study adds to this evidence by demonstrating that emotional demands, measured at the occupational level, are associated with an increased risk of depressive disorder. Our findings suggest that this association cannot be explained by reporting bias or by the selection of individuals with higher pre-employment risk of depression according to the included pre-employment risk factors into occupations with high emotional demands.

### Strengths and limitations

The strengths of this study include its register-based design enabling this large-scale study with annual exposure assessments of emotional demands and a clinical measure for depressive disorder based on hospital treatment data as the outcome. Furthermore, a substantial strength of the study is that we repeated similar analyses in two independent samples of the Danish working population, ensuring the findings’ reproducibility and their generalizability across all ages of the working population.

Our study also entails some limitations. We did not measure emotional demands at the individual level, but used a JEM. While this approach eliminated reporting bias from the study, it had the drawback of possible misclassification of exposure as individuals in occupations with high average levels of emotional demands may not actually be exposed. This misclassification may have led to an under-estimation of the association between emotional demands and depressive disorder (35), as also indicated by the quantitative bias analysis, suggesting that the reported results are conservative estimates of the association between emotional demands and depression. On the other hand, it should be noted that there is also the possibility of an overestimation of the true association between emotional demands and depressive disorder due to measurement bias caused by be systematic group level differences in the reporting of emotional demands, for instance caused by a higher prevalence of depression in occupations with high emotional demands. The magnitude of such bias is difficult to gauge, but its potential existence should be kept in mind when interpreting the results. Furthermore, emotional demands may be related to interactions with clients and customers, or alternatively be related to interactions with colleagues, and these sources may have different mental health effects (36). In the present study, we could not distinguish between the different sources of emotional demands.

Our confounder control was limited to data available from the registers, and we could not control for several risk factors for depression such as genetics, life events, or childhood adversities. However, the adjustment for maternal and paternal psychiatric diagnoses may to some extent have accounted for genetic differences, and both life events and childhood adversities are socioeconomically patterned (37, 38). Consequently, if associations were substantially biased by lack of data on these factors, we would expect associations to be attenuated after adjusting for indicators of childhood socioeconomic position. This was not the case.

Finally, we included only cases of depressive disorder that were hospital diagnosed and the observed associations may differ from those with symptom-based measures (39) as many cases of depressive disorder are untreated or treated exclusively in primary care (40). Results from a European study (40) showed that amongst participants with mood disorder during the past 12 months, there were 36.5% who had consulted a formal health service for their mental health during this period. About one third consulted only their GP. Such cases – in addition to cases who consulted no health service at all – would not be included in our outcome measurement. If there are systematic differences between occupational groups in the likelihood of receiving hospital treatment when depressed, this could lead our study to either over- or underestimate the true association between emotional demands and depressive disorder. Given this limitation, it is important that the results of our study are interpreted in light of other previous studies (eg, 9.) applying outcome measurements that are not affected by treatment seeking behaviors.

### Concluding remarks

This nation-wide register-based study suggests that employees in occupations with high levels of emotional demands are at increased risk of hospital-treated depressive disorder. This increased risk was neither attributable to reporting bias nor explained by the included risk factors for depression recorded before workforce entry.

## Supplementary material

Supplementary material

## Protection of research participants

This study complies with the Declaration of Helsinki. No patients were involved. In Denmark, studies that are based on questionnaire and register-data only do not require approval from the National Committee on Health Research Ethics. The Danish Data Protection Agency approved this study through the joint notification of the National Research Centre for the Working Environment (#2015-57-0074). All data are stored in a protected server environment hosted by Statistics Denmark.
