# Supplementary material for: Emotional demands at work and risk of hospital-treated depressive disorder in up to 1.6 million Danish employees: a prospective nationwide register-based cohort study
Source: Scand J Work Environ Health. 2022 Apr 29;48(4):302–11. doi: 10.5271/sjweh.4020 (PMC9524161; doi:10.5271/sjweh.4020)

Emotional demands at work and risk of hospital-treated depressive disorder in up to 1.6 million Danish employees: a prospective nationwide register-based cohort study[1]

by Ida EH Madsen, PhD,[2] Jeppe Karl Sørensen, MSc,[1] Julie Eskildsen Bruun, MSc,[1] Elisabeth Framke, PhD,[1] Hermann Burr, PhD,[2] Maria Melchior, PhD,[3] Børge Sivertsen, PhD,[4, 5, 6] Stephen Stansfeld, PhD,[7] Mika Kivimäki, FMedSci,[8, 9, 10] Reiner Rugulies, PhD [1, 11, 12]

1. Supplementary material
2. Correspondence to: Ida EH Madsen, National Research Centre for the Working Environment, Lerso Parkalle 105, DK- 2100 Copenhagen, Denmark. [E-mail: ihm@nfa.dk]

Table S1. Measurements of occupation and potential confounders

Variable	Source	Coding
Occupation	Main occupation each year according to Statistics Denmark's Employment Classification Module (AKM). If missing, data included from Register-based Labour Force Statistics (RAS)	DISCO-88
Gender, Age, Cohabitation	Danish Civil Registration System	Man/woman, continuous, yes (living with a partner or spouse) /no (single including children living with their parents) /missing
Employment status	Statistics Denmark's Employment Classification Module ("Arbejdsklassifikationsmodulet")	employed/non-employed

Migration background	Danish Civil Registration System	Yes/no
Income	Statistics Denmark's Income Statistics Register	Deciles
Health services use	Danish National Health services register	Deciles
job control, work-related violence, and physical demands at work	Job exposure matrices based on Danish Work Environment Cohort Study	JEMPAD quartiles, Yes (≥2%)/no, JEMPAD quartiles
Psychiatric disorder before baseline	Psychiatric Central Research Register and National Patient Register. Psychiatric diagnoses coded as chapter F (ICD-10) or 290-315 (ICD-8). Both main and subsidiary diagnoses included	Yes/no
Maternal and paternal employment status	Statistics Denmark's Employment Classification Module	Employed/non-employed
Maternal and paternal education	Statistics Denmark's Student Register	Two separate variables coded according to ISCED: Primary or lower secondary/Upper secondary /Short cycle tertiary/ Bachelor or equivalent/ Master or doctoral/ unknown
Maternal and paternal income	Statistics Denmark's Income Statistics Register	Two separate variables coded as quintiles or unknown

Maternal and paternal psychiatric diagnoses	Psychiatric Central Research Register and National Patient Register. Psychiatric diagnoses coded as chapter F (ICD-10) or 290-315 (ICD-8). Both main and subsidiary diagnoses included	Two separate variables coded yes/no
Maternal and paternal somatic diagnoses, coronary heart disease	I20-I25 (ICD-10) or 410-414 (ICD-8). Both main and subsidiary diagnoses included	Two separate variables coded yes/no
Maternal and paternal somatic diagnoses, cancer	C00-C96 (ICD-10) or 140-195 (ICD-8) or 199-209 (ICD-8). Both main and subsidiary diagnoses included	Two separate variables coded yes/no

Table S2. Measurement of emotional demands in the Danish Work Environment Cohort Study

Item	Response options[a]
Does your work put you in emotionally disturbing situations?	Always; Often; Sometimes; Seldom; Never/hardly ever
Is your work emotionally demanding?	To a very large extent; To a large extent; Somewhat; To a small extent; To a very small extent
Do you get emotionally involved in your work?	To a very large extent; To a large extent; Somewhat; To a small extent; To a very small extent

[a]All items are scored 1 to 5 with the highest values indicating the highest level of emotional demands at work.

Table S3. Occupations with highest and lowest levels of emotional demands

Highest level of emotional demands

**JEMPAD**				**DaWCo**			
**Disco-88 code**	**Name**	**Mean level**	**% observations**	**Disco-88 code**	**Name**	**Mean level**	**% observations**
3443	Government Social Benefits Officials	3.54	0.21	3460	Social Work Associate Professionals	3.48	0.12
3460	Social Work Associate Professionals	3.57	0.17	3443	Government Social Benefits Officials	3.41	0.07
5162	Police Officers	3.29	0.38	2221	Medical Doctors	3.22	0.31
2221	Medical Doctors	3.26	0.56	5162	Police Officers	3.19	0.21
2331	Primary Education Teaching Professionals	3.26	2.83	3231	Nursing Associate professionals	3.13	1.42
3231	Nursing Associate professionals	3.27	1.84	2331	Primary Education Teaching Professionals	3.12	2.15
5160	Protective Services Workers	3.14	0.02	2230	Nursing And Midwifery Professionals	3.09	0.06
3330	Special Education Teaching Associate Professionals	3.2	1.19	3330	Special Education Teaching Associate Professionals	3.09	0.72

2300	Teaching Professionals	3.17	0.23	2460	Religious Professionals	3.08	0.02
2460	Religious Professionals	3.13	0.11	2300	Teaching Professionals	3.08	0.12

Lowest level of emotional demands

**JEMPAD**				**DaWCo**			
**Disco-88 code**	**Name**	**Mean level**	**% observations**	**Disco-88 code**	**Name**	**Mean level**	**% observations**
8260	Textile-, Fur- And Leather-Products Machine Operators	1.54	<0.01	8271	Meat- and Fish-processing-machine Operators	1.26	0.67
8271	Meat- and Fish-processing-machine Operators	1.50	0.64	8332	Earth-moving- and Related Plant Operators	1.28	0.04
8332	Earth-moving- and Related Plant Operators	1.49	0.14	7221	Blacksmiths, hammer-smiths and forging-press workers	1.31	0.35
4110	Secretaries And Keyboard-Operating Clerks	1.69	0.01	8260	Textile-, Fur- And Leather-Products Machine Operators	1.31	0.00
4122	Statistical and Finance Clerks	1.64	0.20	7223	Machine-tool setters and setter-operators	1.35	0.34
8263	Sewing-machine Operators	1.66	0.06	9330	Transport Labourers And Freight Handlers	1.37	1.88

8283	Electronic-equipment Assemblers	1.72	0.19	4122	Statistical and Finance Clerks	1.37	0.16
8290	Other Machine Operators and Assemblers	1.66	0.41	8263	Sewing-machine Operators	1.38	0.03
8270	Food And Related Products Machine Operators	1.64	0.15	8240	Wood-products machine operators	1.39	0.16
9330	Transport Labourers And Freight Handlers	1.63	1.00	8290	Other Machine Operators and Assemblers	1.39	0.41

Table S4. Correlations between included working conditions

	Emotional demands	Job control	Work-related violence	Physical demands at work
**JEMPAD**				
Emotional demands	1.00	0.67	0.58	-0.15
Job control	-	1.00	0.38	-0.32
Work-related violence	-	-	1.00	0.04
**DaWCo**				
Emotional demands	1.00	0.69	0.61	-0.08
Job control	-	1.00	0.41	-0.20
Work-related violence	-	-	1.00	0.13

Table S5. Distribution of individuals according to pre-employment risk factors for depression

	N individuals	%
**Maternal employment status**		
Employed	653987	69.62
Unemployed/nonemployed	192581	20.50
Unknown	92843	9.88
**Paternal employment status**		
Employed	705361	75.09
Unemployed/nonemployed	122213	13.01
Unknown	111837	11.91
**Maternal educational level**		
Primary or lower secondary	207263	22.06
Upper secondary	238061	25.34
Short cycle tertiary	16295	1.73
Bachelor or equivalent	110866	11.80
Master or doctoral	22502	2.40
Not classified/unknown	344424	36.66
**Paternal educational level**		
Primary or lower secondary	123468	13.14
Upper secondary	258121	27.48
Short cycle tertiary	22033	2.35
Bachelor or equivalent	54825	5.84
Master or doctoral	45103	4.80
Not classified/unknown	435861	46.40
**Maternal income**		
1st Quintile	133934	14.26
2nd Quintile	131762	14.03
3rd Quintile	132209	14.07
4th Quintile	132182	14.07
5th Quintile	131916	14.04

Unknown	277408	29.53
**Paternal income**		
1st Quintile	131768	14.03
2nd Quintile	129721	13.81
3rd Quintile	130163	13.86
4th Quintile	129752	13.81
5th Quintile	129461	13.78
Unknown	288546	30.72
**Maternal psychiatric diagnosis**		
Yes	62139	6.61
No	877272	93.39
**Paternal psychiatric diagnosis**		
Yes	57415	6.11
No	881996	93.89
**Maternal somatic diagnosis, coronary heart disease**		
Yes	9348	1.00
No	930063	99.00
**Paternal somatic diagnosis, coronary heart disease**		
Yes	32666	3.48
No	906745	96.52
**Maternal somatic diagnosis, cancer**		
Yes	31158	3.32
No	908253	96.68
**Paternal somatic diagnosis, cancer**		
Yes	23470	2.50
No	915941	97.50
**Missing maternal data**	84202	8.96
**Missing paternal data**	96288	10.25

Table S6. Association between emotional demands and subsequent onset of depression in the JEMPAD and DaWCo cohorts after excluding individuals with any psychiatric diagnosis before baseline

	Hazard Ratio	95% confidence interval	P-value
**JEMPAD** [a]			
**Emotional demands**			<0.001
Low (reference)	1.00		
Medium - low	1.07	1.02 - 1.13	
Medium - high	1.03	0.97 - 1.10	
High	1.34	1.25 - 1.44	
**DaWCo** [b]			
**Emotional demands**			<0.001
Low (reference)	1.00		
Medium - low	0.94	0.88 - 1.01	
Medium - high	1.20	1.12 - 1.29	
High	1.29	1.14 - 1.45	

[a] We report fully adjusted estimates, i.e. for JEMPAD associations are adjusted for: gender, age, cohabitation, employment status, migration background, income, health services use, job control, work related violence and physical demands at work.

[b] We report fully adjusted estimates, i.e. for DaWCo associations are adjusted for gender, age, cohabitation, employment status, migration background, income, health services use, job control, work related violence and physical demands at work, maternal and paternal employment status, education, income, and maternal and paternal psychiatric and somatic diagnoses.

Figure S1. Directed Acyclic Graph of the analyzed associations

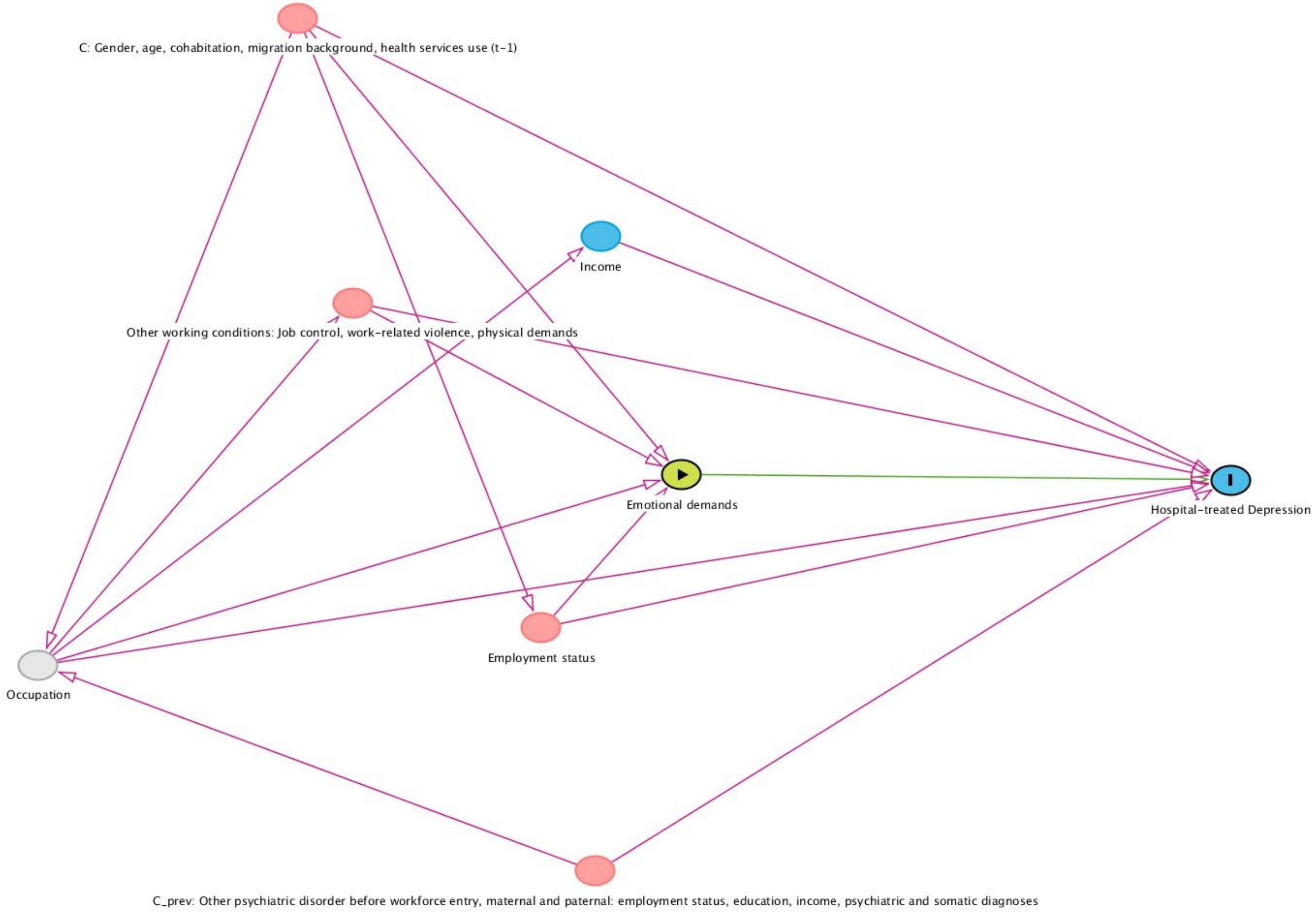

Supplement: Supplementary material [file SJWEH-48-302-S001.pdf]
